# Improving the Efficiency of Colchicine-Based Chromosomal Doubling of Maize Haploids

**DOI:** 10.3390/plants9040459

**Published:** 2020-04-05

**Authors:** Vijay Chaikam, Manje Gowda, Leocadio Martinez, John Ochieng, Hamilton Amoshe Omar, B.M. Prasanna

**Affiliations:** 1 International Maize and Wheat Improvement Center (CIMMYT), ICRAF campus, UN Avenue, Gigiri, Nairobi P.O. Box 1041–00621, Kenya; V.Chaikam@cgiar.org (V.C.); M.Gowda@cgiar.org (M.G.); J.Ochieng@cgiar.org (J.O.); H.Omar@cgiar.org (H.A.O.); 2 International Maize and Wheat Improvement Center (CIMMYT), Mexico D.F. 6-641 06600, Mexico; L.Martinez@cgiar.org

**Keywords:** maize, haploid, chromosome doubling, colchicine, doubled haploid

## Abstract

Production and use of doubled haploids (DH) is becoming an essential part of maize breeding programs worldwide as DH lines offer several advantages in line development and evaluation. One of the critical steps in maize DH line production is doubling the chromosomes of in vivo-derived haploids so that naturally sterile haploids become reproductively fertile diploids (DH) to produce seed. This step of artificially doubling the chromosomes is labor-intensive and costly; hence, optimizing protocols to improve the doubling success is critical for achieving efficiencies in the DH production pipelines. Immersion of 3–4-day old germinating haploid seedlings in colchicine solution is commonly used for chromosome doubling in large-scale maize DH line production. This manuscript presents a new method of colchicine application to haploid seedlings that showed superior doubling rates compared to other methods like standard seedling immersion, seed immersion, root immersion, and direct application of colchicine solution to the seedlings at V2 stage in the greenhouse trays. The new method involves immersing the crown region of the haploid seedlings along with all the seedling roots at V2 stage in the colchicine solution. Further experiments to optimize this method indicated that increasing colchicine concentration had a very positive effect on overall success rate in chromosomal doubling, while not drastically affecting survival rate. The optimized method showed on average 5.6 times higher overall success rate (OSR) compared to the standard haploid seedling immersion method which was the second-best method in our experiments. This improved method of colchicine application saves resources by reducing the number of haploids to be generated and handled in a maize DH production pipeline.

## 1. Introduction

Doubled haploids (DH) are an efficient alternative to conventional inbred lines developed through recurrent selfing in maize breeding. Compared to conventional inbred lines, DH lines facilitate faster variety registration and intellectual protection due to their distinctness, uniformity, and stability, thereby supporting quicker deployment of maize hybrids in the market [1,2]. Higher genetic variance and higher heritabilities when using DH lines compared to conventional selfed lines in per se and testcross evaluation facilitate accurate selection decisions. The major steps in maize DH line production pipelines are in vivo induction of haploids, separation of haploids from diploids, doubling the genome in haploid (D_0_) plants, and production of D_1_ seed from D_0_ plants [3,4]. Development of protocols for large-scale DH line production and continuous process improvement in the last three decades resulted in higher efficiencies at different steps of DH line development [3]. 

DH production in maize relies on in vivo haploid induction using maternal haploid inducers. Maternal haploid inducers with high haploid induction rates were developed in both temperate and tropical genetic backgrounds making large-scale induction of haploids possible from diverse maize germplasm in a cost-efficient manner [2,5,6]. Haploids are separated from diploids based on phenotypic markers integrated in the inducers, like *R1-nj* [7,8], red root [6,9], and high oil [10]. Recently, efforts were put into automation of haploid identification process using high oil [11,12] and *R1-nj* markers [13,14,15,16] that can lead to significant resource savings. 

Chromosomal doubling is essential for restoring fertility and production of seed from haploids. However, improving efficiency in the chromosomal doubling process is relatively less researched/published compared to other steps in the DH production pipeline. Spontaneous chromosomal doubling occurs in too low frequencies in most maize genotypes [17,18]. Therefore, chromosomal doubling by treating the haploids with mitosis inhibiting chemicals is commonly practiced [19,20]. Chromosome doubling protocols involves several labor-intensive steps and use of expensive chemicals. Hence, increasing chromosomal doubling efficiency is critical to reduce the cost per DH line [3].

Commonly reported chemicals for achieving artificial chromosome doubling in maize haploids include colchicine [21,22,23], antimitotic herbicides [20], and nitrous oxide (N_2_O) gas [24,25]. Colchicine is much more toxic compared to N_2_O gas and anti-mitotic herbicides [20,25]. However, colchicine is more commonly used in DH production pipelines because the colchicine is more readily available for purchase in large quantities and protocols are well-established. Several methods of colchicine application to haploids were described in literature that include (a) treating the seed; (b) immersing germinating haploid seedlings in colchicine solution; (c) immersing the roots of haploid seedlings; or (d) injecting colchicine into shoot apical meristem [3]. Among these, the seedling immersion method is widely adapted for large-scale DH line production as it showed a better success rate compared to seed treatment [23], root treatment [22], or injection into shoot apical meristem [26]. However, there is no publication showing a robust comparison of maize haploid chromosome doubling efficiencies of these methods as most studies used small sample sizes of haploids and different criteria to assess doubling efficiencies. Therefore, the objectives of the present study were (a) to systematically evaluate chromosomal doubling success rates in five different methods of colchicine application to maize haploids; and (b) to further optimize the colchicine application method to achieve higher success rates. 

## 2. Results

### 2.1. Comparison of Doubling Efficiencies in Different Methods of Colchicine Applications

In Experiment 1, assessment of success rates associated with three different methods of colchicine application to maize haploids revealed that Method II involving root and crown immersion in 0.04% colchicine and 0.1% dimethyl sulfoxide (DMSO) at V2 stage showed higher survival rate (SR), reproduction rate (RR), and overall success rate (OSR) compared to other methods of colchicine application (Table 1). OSR was about 1.5 times higher in Method II compared to Method I which is commonly used in DH production pipelines and comprises of immersing 2–3-day old germinating seedlings. Method III involving treating roots and coleoptile region of V2 seedlings without removing them from greenhouse trays did not show significant difference in SR, RR, and OSR compared to the control treatment that had no colchicine (Table 1). In Experiment 2, Method IV involving seed immersion in different concentrations of colchicine and 0.1% DMSO solution after 20 h of imbibition in water was tested in comparison to Method II. Method IV had significantly lower RR and OSR than Method II and the rates were not significantly different from the control. Increasing the concentration of colchicine from 0.05% to 0.3% did not result in a significant increase in RR and OSR in Method IV (Table 2). SR in Method IV at higher concentrations was not significantly different from that obtained by Method II (Table 2). In Experiment 3, Method V involving immersion of roots only until half of their length from root tips was compared with Method II. Method V showed significantly lower RR and OSR compared to Method II and these rates were not significantly different from the control (Table 3). SR was also not significantly different between Methods V and II (Table 3). Method II showed similar OSR in Experiments 1, 2, and 3 (Table 1, Table 2 and Table 3). SR for all treatments and controls in Experiments 1 and 2 was notably low compared to SR recorded in all other experiments. This was due to high incidence of maize lethal necrosis (MLN) in our nurseries when these experiments were conducted.

### 2.2. Optimization of Method II

As OSR was the highest in Method II among all the five methods tested, we further explored the opportunity to improve the OSR through this method. In Experiment 4, we compared the survival rates in direct transplanting in the field after colchicine treatment versus transplanting of the treated seedlings after recovery in the greenhouse. There was a significant increase in the SR when seedlings were directly transplanted compared to transplanting after recovery in greenhouse (Table 4). Hence, in subsequent experiments, we used direct transplanting in the field without recovery in greenhouse. In Experiment 5, the effect of increasing DMSO concentration on chromosomal doubling was evaluated (Table 5). Even though there was a positive trend in increasing OSR with increase of DMSO concentration from 0.1% to 0.5%, it was not statistically significant. SR and RR were also not significantly different at different concentrations of DMSO. However, OSR was significantly different at all concentrations of DMSO compared to the control that had no DMSO. In Experiment 6, effect of different colchicine concentrations on doubling was evaluated (Table 6). When colchicine concentration was increased from 0.04% to 0.1%, SR decreased significantly but RR and OSR were more than doubled. However, SR, RR, and OSR were not significantly different at colchicine concentrations of 0.07% and 0.1%.

### 2.3. Validation of Improved Method II in Large-Scale DH Production Pipeline

To ascertain the effectiveness of improved Method II across multiple populations, a total of 36,000 haploids from 12 populations were used in Experiment 6 that compared improved Method II versus the commonly used Method I (Table 7). Method II showed consistently very high RR and OSR in all the populations compared to Method I. On average, RR and OSR were 5.5 and 5.3 times higher, respectively, in Method II compared to Method 1. SR was not significantly different between the two methods. In both methods, RR and OSR varied greatly depending on the population. In Method I, RR ranged from 3.1 to 11.7 while it ranged from 24.9 to 61.5 in Method II. OSR in Method I ranged from 2.7 to 10.5 while it ranged from 23.5 to 58.5 in Method II. 

**Figure 1:**
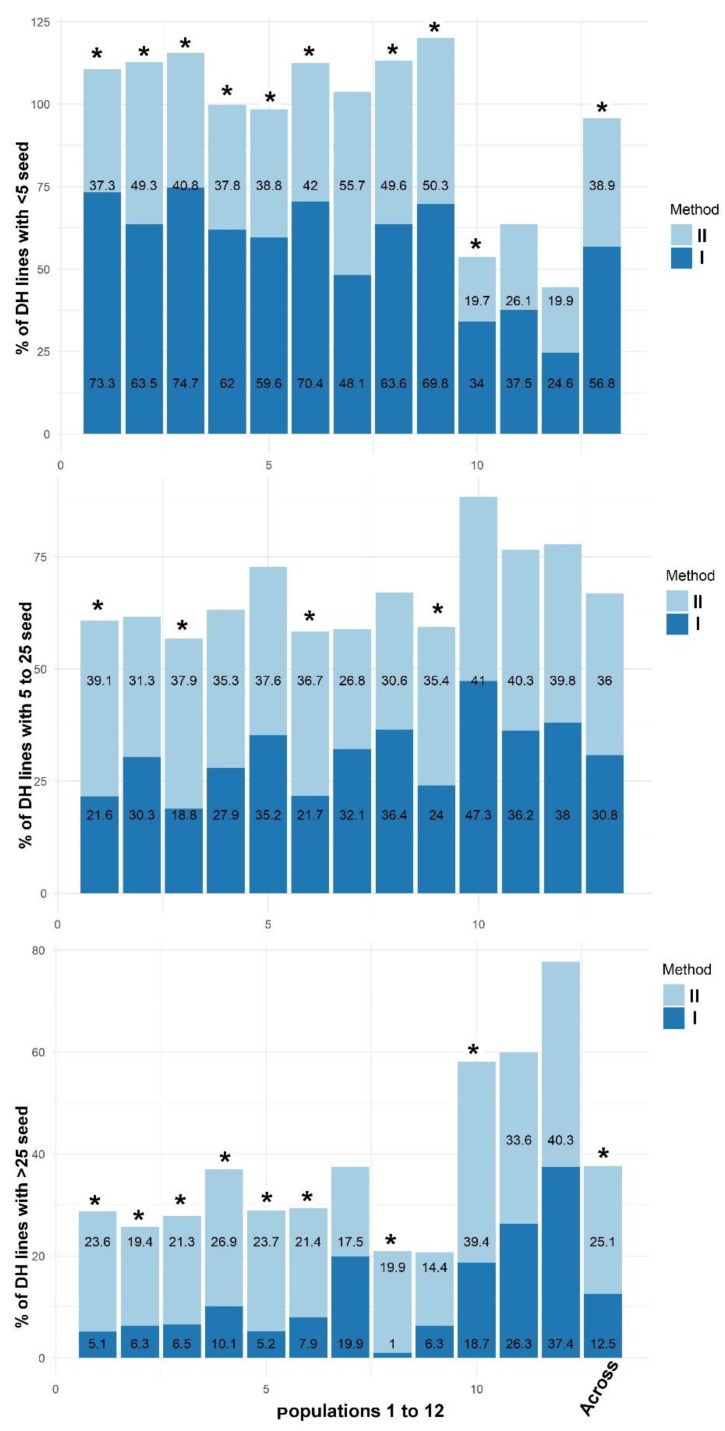
Comparison of seed quantity among D_1_ ears generated by using Method II vs. Method I. In the graphs, * indicates statically significant differences among the two methods tested for proportion of doubled haploids (DH) lines in each category.

In addition to rates, seed quantity on DH ears resulting from each method was analyzed (Figure 1). A total of 4339 and 791 DH lines were obtained from haploids subjected to Method II and Method I, respectively. Across populations, the proportion of DH lines containing less than five seeds was almost 1.5 times less when using Method II compared to Method I. Among the 12 populations, nine populations showed a significantly lower proportion of DH lines with less than five seeds when using Method II compared to Method I. Additionally, the proportion of DH lines having more than 25 seeds across populations was more than double in Method II compared to Method I. Among the 12 populations, eight populations showed a significantly higher proportion of DH lines with >25 seeds from Method II compared to Method I. There was no significant difference in proportion of DH lines having 5–25 seeds among the two methods. In summary, Method II resulted in greater recovery of DH lines (D_1_ ears) with higher seed quantity compared to Method I.

## 3. Discussion

Chromosome doubling is the most intricate step in maize DH line production as it involves several critical activities like germination of haploids, preparation and treatment of haploid seedlings with mitosis inhibiting chemicals, post-treatment recovery, and transplanting in the field [19]. Hence, achieving higher success rates in chromosomal doubling could lead to higher efficiencies in DH production pipelines. Protocols used for doubling chromosomes in maize haploids were developed in the 1990s and involve application of colchicine to seed, whole germinating seedlings or roots, or the shoot apical meristematic region of 8–12-day old seedlings [21,22,27]. A systematic comparison of doubling efficiencies among different colchicine-based chromosome doubling protocols based on large enough samples and replicated experiments was lacking in literature. Additionally, there was no uniform criteria used to assess chromosomal doubling efficiency of different protocols. Some studies considered chromosomal counts through microscopic studies [21], some considered pollen production [23], while others considered seed production [26] as indicators of success of a chromosome doubling treatment of maize haploids. Most studies did not consider mortality as a result of toxicity of chromosome doubling chemicals in determining the success. Melchinger et al. [20] proposed three parameters namely SR, RR, and OSR to assess the success in chromosomal doubling, as these parameters sufficiently consider all the aspects of the chromosomal doubling step. These parameters were used to assess the efficiency of chromosomal doubling using antimitotic herbicides and N_2_O gas in comparison to the standard method of immersing germinating seedlings in colchicine solution [20,25]. Among the three parameters, OSR was found to be particularly important for maize DH production pipelines as it considers all the factors important for seed production from haploids. 

In this study, the criteria described by Melchinger et al. [20] were used to assess chromosomal doubling success through five different methods of colchicine application using replicated trials and large sample sizes. The study revealed that immersion of haploid seedlings from root tips up to 2–3 cm above seed level at V2 growth stage (Method II) is the most effective method in doubling the chromosomes. This method resulted in more than double the OSR compared to standard seedling immersion method at VE stage (Method I). At V2 stage if seedlings were immersed in colchicine from root tips up to half the length of roots (Method V), it resulted in significantly lower OSR than immersing the seedlings until 2–3 cm above the seed level. Previous studies that compared immersion of roots only with standard seedling immersion method using colchicine [22] and antimitotic herbicides [20] showed very low success for root immersion compared to seedling immersion. Together, immersion of the crown region encompassing the shoot apical meristem along with roots appears to be essential to achieve higher levels of OSR. The seedling immersion method (Method I) used in this study was slightly different from seedling immersion method used earlier [20,25] in that the seedlings were treated in a lower concentration of colchicine (0.04% vs. 0.06%) and for much longer time (12 h vs. 8 h). Earlier studies [20,25] reported an OSR of ≈10% for the seedling immersion method in the majority of the experiments. Lower OSR for this method in our experiments could be the result of using a lower concentration of colchicine. 

Our experiments also revealed that application of colchicine directly to seedling trays in greenhouse (Method III) and to imbibed seed (Method IV) were very inefficient in causing chromosomal doubling. A previous report on seed treatment by Chalyk [23] also indicated that seed treatment did not result in chromosomal doubling. However, another report by Gayen et al. [21] indicated that when seeds were treated with 0.06% colchicine with a portion of plumule cut or not cut, it showed higher doubling rates than treating germinating seedlings at the same colchicine concentration. However, the sample sizes of 21 and 50 seeds in these two earlier studies [23] and [21], respectively were inadequate to arrive at meaningful conclusions compared to the present study with large sample sizes.

Since Method II involving immersion of whole roots and crown region was found to be more efficient in doubling haploid chromosomes, we tested if increasing concentrations of colchicine and DMSO could further increase OSR. Increasing DMSO concentration from 0.1% to 0.5% led to a slight but nonsignificant increase in OSR. However, increasing colchicine concentration had a very strong positive effect on OSR. It may be possible to achieve even higher doubling rates in Method II than what is reported in this study by further increasing colchicine concentrations. When evaluated in multiple populations, Method II consistently showed significantly higher SR, RR, and OSR than Method I in all the 12 populations studied. In both Methods I and II, OSR varied among the populations. Earlier studies also pointed to genotypic influence on artificial doubling of haploid chromosomes [18] or spontaneous chromosomal doubling [17,28,29].

The improved method of immersing seedling roots and crown region in 0.07%–0.1% colchicine and 0.5% DMSO is the most efficient method of chromosomal doubling reported so far. This method has several advantages compared to the currently used seedling immersion method (Method I). As OSR is several-fold higher in Method II compared to Method I, use of Method II in DH pipelines, as reported in this study, could result in significant resource savings. For example, field space in induction nursery, greenhouse space, and field space in D_0_ nursery can be reduced significantly as the number of haploids to be generated and handled will be much lower through Method II. Currently, when using Method I, significantly high labor costs are incurred as it involves labor-intensive steps like seed germination in paper towels, checking for germination for 3–4 days, cutting the shoot tips, doubling treatment, potting in greenhouse, caring in greenhouse for 8–12 days, and transplanting in the field. As Method II involves relatively fewer steps like germination in greenhouse trays, caring for seedlings for 8–12 days, doubling treatment followed by direct transplanting in the field, relatively less labor is needed. In addition, it is much easier for people to handle relatively bigger seedlings at V2 stage in Method II than handling germinating seedlings in Method I. Higher success rate in Method II also facilitates application of genomic technologies like genomic selection at haploid stage that leads to delivery of DH lines with desirable haplotypes. In addition, increased seed quantity on DH ears from Method II also facilitates phenotypic and genotypic selection on the majority of DH lines produced without needing an additional cycle of seed increase. However, the increased concentrations of colchicine and DMSO in Method II compared to Method I, will increase the cost of chromosomal doubling treatments but overall DH line production cost might not be significantly affected as the cost of chemicals is a much smaller contributor to the overall DH production costs [20]. 

Despite the benefits outlined above, it must be noted that colchicine is very toxic to humans [20,30]. Hence it is desirable to use chemicals that are less toxic. Recently, efforts to develop much safer protocols indicated that it is possible to achieve similar levels of chromosomal doubling efficiencies as colchicine in Method I when using anti-mitotic herbicides and N_2_O gas [20,25]. However, there are still limitations on using safer alternatives like availability and cost of these chemicals. For example, mitotic herbicides like amiprofos-methyl (APM) and pronamide are reported to result in OSR similar to colchicine application as in Method I [20]. However, both chemicals are not registered for sale as herbicides in most countries (e.g., in Africa and Asia) and hence are not widely available. Pure laboratory grade chemicals are more costly than colchicine and are available only in limited quantities. Use of N_2_O gas requires a specialized gas chamber which may be of significant initial establishment cost [24,25]. Considering these limitations with non-colchicine-based alternatives, Method II described here could provide a practical solution until the limitations with colchicine alternatives are effectively addressed. Irrespective of the chemical used for doubling haploid chromosomes, it is important to follow adequate occupational health and safety measures. 

## 4. Materials and Methods

### 4.1. Genetic Materials and Experimental Locations

In Experiments 1–4 (described in subsequent section), haploids derived from 20 CIMMYT maize populations adapted to mid-altitude/subtropical regions of eastern Africa were used. Haploids were induced in the summer cycle of 2015 as described earlier [31] using a tropical inducer TAIL9 × TAIL8 [9] at CIMMYT’s Maize Doubled Haploid Facility at Kiboko, Kenya (2.25° S, 37.73° E). Putative haploid kernels were identified based on *R1-nj* (Navajo) anthocyanin marker expression on kernels. A set of 1500 putative haploid kernels were taken from each of the induced populations and a balanced bulk was constituted. Required samples sizes were drawn from this balanced bulk and used in experiments carried out at the Maize Doubled Haploid Facility at Kiboko, Kenya. Experiment 1 was carried out in the winter season of 2016; Experiments 2 and 3 were undertaken in summer season of 2017, and Experiment 4 was implemented in winter season of 2018.

For Experiments 5 and 6, haploids derived from 13 CIMMYT maize populations adapted to the subtropics and four populations adapted to the lowland tropics of Mexico were used. Haploids were induced in the winter season of 2017 as described above at CIMMYT’s Agua Fria experimental station (20.26° N, 97.38° W) in the state of Puebla, Mexico, using tropical hybrid inducer CIM2GTAIL006 × CIM2GTAIL009 [5]. Putative haploid kernels were identified based on *R1-nj* marker expression. A set of 1000 haploids were sampled from each population and mixed to make a balanced bulk. Experiments 5 and 6 were carried out in the winter cycle of 2018 at Metztitlan (20.6° N, 98.76° W) in the state of Hidalgo, Mexico. 

For Experiment 7, haploids derived from nine subtropical populations and three tropical lowland populations were used. Haploids were induced in summer season of 2017 at Agua Fria. Haploid induction and identification were carried out as described in Experiments 4 and 5. This experiment was carried out in the winter cycle of 2019 at Agua fria experimental station. 

All experiments were conducted in randomized complete block design where replications from each treatment were blocked.

### 4.2. Details of Experiments

#### 4.2.1. Experiment 1

Three different methods of colchicine application were compared for their doubling efficiency. A set of 3000 putative haploid seeds were used to test the doubling efficiency of each method. The experiment was carried out in three replications for each method with 1000 seeds per replication. Method I involving germination of haploid seed in paper towels, immersing germinating seedlings in 0.04% colchicine + 0.1% DMSO after cutting the coleoptile tip was carried out as described by Chaikam and Mahuku [19]. Treated seedlings were cared for in greenhouse and field as described by Mahuku [32]. In Method II, seedlings were germinated in 96-well plastic greenhouse trays with volcanic cinder (gravel) of particle size ≈ 2 mm in a greenhouse. Seedlings were grown for 10–12 days until they reached V2 stage. Seedlings were sprayed with foliar nutrients 6 days after planting. Just before the treatment, seedlings were pulled out carefully from volcanic cinder to avoid root damage and washed in a plastic tub with tap water to remove the medium. Washed seedlings were aligned at seed level, bundled and placed in 2-L plastic round containers. Each container holds 120–130 seedlings. Then, 0.04% colchicine and 0.1% DMSO solution was poured into the beaker from one side until all the seedling roots and coleoptile region 2–3 cm above the seed level were immersed in the solution. Seedlings were maintained in colchicine solution in the lab at ambient temperature for 5 h. Spent colchicine solution was collected in a plastic tank for safe disposal. The beakers with seedlings were filled with tap water, gently rinsed, and the water was emptied into a plastic container for disposal. Later, seedlings were washed three times in a sink with tap water. Seedlings were replanted in trays in volcanic cinder and maintained for another 8 days for recovery. Seedlings were sprayed with foliar nutrients 2 days after potting in greenhouse. After recovery, seedlings were transplanted in a well irrigated field and provided good agronomic management as described by Mahuku [32]. 

For colchicine application by Method III, seedlings were grown in a greenhouse for 10–12 days until they reached V2 stage as described above. In the greenhouse, one of the benches was lined up with a thick polythene sheet on the bottom and all sides to create a tub that can hold seedling trays. This tub had a depth of approximately twice the height of seedling tray. Seedling trays were placed in the tub and the tub was filled with 0.04% colchicine + 0.1% DMSO solution until the collar was immersed in colchicine for 5 h. After the treatment, colchicine was emptied by sucking the solution with a pump into a plastic container. Tap water was applied to the tub and then the tub and trays with seedlings were washed three times. Seedlings were maintained in the trays for another 8 days and the seedlings were transplanted in the field and managed in the field as described for Method II. In this experiment, control treatment was seedling immersion method treated with tap water instead of colchicine and DMSO solution.

#### 4.2.2. Experiment 2

In this experiment, Method II (as described above) was compared to the seed immersion method (Method IV). Method IV was adapted from Gayen et al. [21]. A set of 600 putative haploid seeds were used for each treatment, with each treatment having three replications of 200 seeds each. The seeds were placed in mesh bags and the bags were kept in beakers with tap water at room temperature and imbibed for 20 h. After imbibition, mesh bags with seeds were placed in colchicine solution for 5 h. Five different concentrations of colchicine were tested in Method IV ranging from 0.05% to 0.3% colchicine. DMSO was used at a concentration of 0.1% in all the treatments. After the treatment, seeds were washed three times under tap water, and then directly planted in the field. Method II was carried out as described in Experiment 1. The control experiment was same as Method II except that only tap water was used for treatment instead of colchicine + DMSO solution.

#### 4.2.3. Experiment 3

In this experiment, Method II was compared against Method V involving immersion of only root tissues in colchicine solution. A set of 600 putative haploid seeds were used for each treatment, with each treatment having three replications with 200 seeds per replication. In Method V, seedlings were germinated and grown as described for Method II in Experiment 1. Seedlings were immersed at half the length of the roots from root tips in 0.04% colchicine and 0.1% DMSO solution for 5 h. Method II was carried out as described in Experiment 1. The control experiment was same as Method II except that only tap water was used for treatment instead of colchicine + DMSO solution.

#### 4.2.4. Experiment 4

To determine if seedling recovery in a greenhouse is required after chromosomal doubling treatment in Method II, seedlings were grown in a greenhouse and were subjected to doubling treatment as described in Method II in Experiment 1. For each treatment, a set of 2100 putative haploid seeds was used. The experiment was conducted in three replications with each replication having 700 seeds. Treated seedlings were either directly transplanted or transplanted after 8 days of recovery in the greenhouse. The control experiment was same as Method II except that only tap water was used for treatment instead of colchicine + DMSO solution and the seedlings were transplanted after 8 days of recovery in greenhouse.

#### 4.2.5. Experiment 5

To test if increasing DMSO concentration has any effect on success rates in colchicine-based chromosomal doubling, seedlings were subjected to Method II as described in Experiment 1. Colchicine concentration of 0.04% was used in all the treatments. Three DMSO concentrations (0.1%, 0.25%, 0.5%) were tested. A control without adding DMSO was included. All the seedlings were directly transplanted to the field after treatment. For each treatment a set of 600 putative haploid seeds was used and experiment was conducted in three replications with each replication consisting of 200 seeds.

#### 4.2.6. Experiment 6

To test if altering colchicine concentration has any effect on chromosomal doubling, seedlings were subjected to Method II as described in Experiment 1. Three concentrations of colchicine (0.01%, 0.04%, 0.07%, 0.1%) were tested and in all experiments; the treatment included 0.1% DMSO. A control with no colchicine was included. All the seedlings were directly transplanted to the field after treatment. For each treatment a set of 600 putative haploid seeds was used. The experiment was conducted in three replications with each replication consisting of 200 seeds.

#### 4.2.7. Experiment 7

To ascertain the effectiveness of Method II compared to Method I across multiple populations in a large DH production pipeline, haploids derived from 12 populations were subjected to both methods. In Method II, 0.1% colchicine and 0.5% DMSO was used. Method I was as described in Experiment 1. From each population, a set of 1500 putative haploid seeds was used for Method I and another set of 1500 seeds was used for testing Method II. Each method was tested in two replications and 750 seeds per population was used in each replication. Together, a total of 18,000 putative haploids were subjected to Method I across populations and an equal number subjected to Method II. Seed quantity in resulting DH ears from both methods was also compared in this experiment.

### 4.3. Data Collection and Analysis

In all the experiments except in Experiment 4, the following data were recorded: (1) number of haploid seeds used for experiment; (2) number of seeds germinated; (3) number of seeds/seedlings subjected to treatment; (4) number of seedlings potted in the greenhouse; (5) number of seedlings transplanted in the field; (6) number of D_0_ plants surviving at pollination; (7) number of D_0_ plants pollinated; (8) number of D_0_ plants that produced D_1_ seed. In Experiment 4, data on D_0_ plants that were pollinated and plants that produced seed was not recorded. The SR, RR, and OSR were calculated as described in [20] using the following formulae, and were expressed in %:

SR = number of plants surviving at pollination/number of seeds or seedlings subjected to treatment; 

RR = number of D_0_ plants that produced seed/number of surviving D_0_ plants at pollination;

OSR = number of D_0_ plants that produced seed/ number of putative haploid seeds or seedlings subjected to chromosome doubling treatment.

In all experiments, except Experiment 2, seed germination and seedling survival rates before chromosomal doubling treatments were not considered in calculating the rates as these losses are not the result of doubling treatments per se. In Experiment 2, for Method IV, seedling germination was considered as the method involved treating the haploid seeds. False positives were also not considered in analyzing the data as they occur at uniform rates in all the treatments. 

In Experiment 7, for analysis of seed quantity resulting from fertile D_0_ plants, the D_1_ ears obtained were categorized into (1) ears having less than five seeds; (2) ears having 5–25 seeds; and (3) ears having more than 25 seeds. Proportion of D_1_ ears in each category was obtained by dividing the number of ears in that category by total number of ears. Proportions in each category obtained in Method I were compared to proportions recorded in Method II.

In all experiments, statistical analyses of variance were performed using PROC GLIMMIX procedure in SAS version 9.2 (SAS Institute, Cary, NC, USA). 

## 5. Conclusions

Improving the success rates in chromosome doubling of maize haploids can result in significant cost savings in line development. Previous reports on chromosomal doubling methods based on colchicine were constrained with small sample sizes and lack of uniform criteria to assess success rates. This study presents a comprehensive analysis of success rates obtained in five different methods of chromosomal doubling based on colchicine using uniform criteria and large enough samples. The analysis revealed that a new method involving immersing the crown region of the haploid seedlings along with all the roots at V2 stage results in high success rates compared to all other methods tested. This method was further optimized, and the improved method was validated in multiple populations in comparison to the commonly used seedling immersion method. Improved crown and root immersion method consistently resulted in higher success rates compared to the standard seedling immersion method in multiple populations. In addition to improved success rates, the crown and root immersion method offers other benefits to DH production pipelines like operational simplicity and reduced labor requirement.

## Figures and Tables

**Table 1:** Comparison of success rates derived from Methods I, II, and III of colchicine application to maize haploids.

Method of Colchicine Application	Target Tissues/Organs	Stage	Colchicine Concentration (%)	N	SR (%)	RR (%)	OSR (%)
Method I	Whole seedling	VE	0.040	3000	47.11b	12.56b	5.94b
Method II	Root and crown region	V2	0.040	3000	63.50a	16.87a	10.67a
Method III	Root and crown region	V2	0.040	3000	52.32b	2.54c	1.32c
Control *	Whole seedling	VE	0.00	3000	71.37a	2.00c	1.42c

N = number of putative haploids used; SR = survival rate; RR = reproduction rate; OSR = overall success rate; letters a, b, and c indicate if the rates are significantly different among different methods. The values with the same letter are not statistically significantly different. * Control = treatment as in Method II, but with colchicine and DMSO solution was replaced by tap water.

**Table 2:** Comparison of success rates from Methods IV and II of colchicine application to maize haploids.

Colchicine Application	Target Tissue/Organs	Stage	Colchicine Concentration	N	SR	RR	OSR
Method IV	Seed	Seed	0.050	600	81.34a	2.23b	1.83bc
			0.075	600	72.50ab	4.45b	3.34b
			0.100	600	70.00abc	1.40b	1.00c
			0.200	600	56.67abc	0.87b	0.50c
			0.300	600	48.17bc	1.71b	0.67c
Method II	Root and crown region	V2	0.040	600	40.33c	30.32a	11.90a
Control *	Root and crown region	V2	0.000	600	73.20ab	0.99b	0.69c

N = number of putative haploids used; SR = survival rate; RR = reproduction rate; OSR = overall success rate; letters a, b, and c indicate if the rates are significantly different among different methods. The values with the same letter are not statistically significantly different. * Control = treatment as in Method II, but with colchicine and DMSO solution was replaced by tap water.

**Table 3:** Comparison of success rates recorded from Methods V and II of colchicine application to haploid seedlings. Control was treatment as in Method II but colchicine was replaced by tap water.

Method	Target Tissue/Organs	N	SR	RR	OSR
Method II	Root and crown region	600	90.40a	11.42a	10.33a
Method V	Roots	600	92.33a	3.96b	3.67b
Control *	Root and crown region	600	92.10a	0.76b	0.58b

N = number of putative haploids used; SR = survival rate; RR = reproduction rate; OSR = overall success rate; letters a and b indicate if the rates are significantly different among different methods. The values with the same letter are not statistically significantly different. * Control = treatment as in Method II, but with colchicine and DMSO solution was replaced by tap water.

**Table 4:** Comparison of survival rates of treated seedlings from Method II involving different transplanting methods in the field.

Method	Transplanting Method	N	SR
Method II	Transplanting after recovery	2100	83.7b
Method II	Direct transplanting	2100	92.5a
Control *	Transplanting after recovery	2100	93.0a

N = number of putative haploids used; SR = survival rate; letters a and b indicate if the rates are significantly different among different methods. The values with the same letter are not statistically significantly different. * Control = treatment as in Method II, but with colchicine and DMSO solution was replaced by tap water.

**Table 5:** Effect of different concentrations of dimethyl sulfoxide (DMSO) on success rates through Method II.

DMSO Concentration (%)	N	SR	RR	OSR
0.5	600	89.67a	18.36a	16.50a
0.25	600	87.50a	13.91ab	12.17ab
0.1	600	89.00a	13.34ab	11.83ab
Control *	600	90.50a	10.36b	9.33b

N = number of putative haploids used; SR = survival rate; RR = reproduction rate; OSR = overall success rate; letters a and b indicate if the rates are significantly different at different DMSO concentrations. The values with the same letter are not statistically significantly different. * Control = treatment as in Method II, but with no DMSO.

**Table 6:** Effect of different concentrations of colchicine on success rates in Method II.

Colchicine Concentration (%)	N	SR	RR	OSR
0.10	600	88.66b	35.76a	31.83a
0.07	600	89.00b	31.10a	27.67a
0.04	600	94.17a	16.00b	15.00b
0.01	600	95.00a	2.63c	2.50c
Control *	600	93.50a	3.40c	3.17c

N = number of putative haploids used; SR = survival rate; RR = reproduction rate; OSR = overall success rate; letters a, b, and c indicate if the rates are significantly different at different colchicine concentrations. The values with the same letter are not statistically significantly different. * Control = treatment as in Method II, but with no colchicine.

**Table 7:** Validating the efficiency of Method II versus Method I across multiple populations.

Population	N	SR		RR		OSR	
		Method II	Method I	Method II	Method I	Method II	Method I
(((CL106653/PHK29)-B/P501SYN))	3000	80.0a	89.6b	38.4a	8.4b	30.9a	7.5b
(((CL106726/LH185)-B/CL420801//P502SYN2))	3000	85.1a	88.2a	39.4a	9.9b	33.5a	8.7b
(((CML321/PHK56//CLWN247)/P502SYN1))	3000	84.6a	83.8a	38.2a	5.8b	32.4a	4.8b
(((CML373/PHHB9)-B/CLHP0005)-B)	3000	91.1a	86.2a	36.2a	3.1b	32.8a	2.7b
(((CML373/PHP38//CML311)/P501SYN))	3000	84.7a	89.2a	38.2a	10.2b	32.5a	9.0b
(((CML384/LH185)-B/CL420801)-B)	3000	94.7a	87.9b	24.9a	5.0b	23.5a	4.4b
(((CML384/PHJ89//CLWN247)/P502SYN1))	3000	86.2a	91.1a	42.7a	3.4b	37.1a	3.1b
(((CML545/LH194)-B/CLHP0049)-B)	3000	91.9a	78.1b	29.3a	5.0b	26.9a	3.9b
(((DTPYC9F46 × LPSC7F64DH:48) × CML551)/((DTMA-103 × LPSC7F64DH:37) × CLWN247))	3000	84.7a	75.9b	55.7a	10.3b	47.1a	7.9b
(((LaPostaSeqC7F711212BBBBCL04934)DH57 × CML551)/((LaPostaSeqC7F711212BBBBCL04934)DH46 × CLWN247))	3000	91.6a	90.3a	57.0a	6.0b	52.3a	5.3b
((CML78/PHP38//CML311/P501SYN))	3000	83.1a	91.6b	36.6a	11.4b	30.5a	10.5b
(LPSC7F64/DTPWC9F115)	3000	95.2a	84.9b	61.5a	11.7b	58.5a	9.9b
Means		87.7a	86.4a	41.5a	7.5b	36.5a	6.4b

N = number of putative haploids used; SR = survival rate; RR = reproduction rate; OSR = overall success rate; letters a and b indicate if the rates are significantly different among the two methods tested. The values with the same letter are not statistically significantly different.
